# The effect of diagnosis-related group policy on treatment cost and treatment efficiency of inpatients with coronary heart disease in Xinjiang: an interrupted time series analysis

**DOI:** 10.7189/jogh.16.04078

**Published:** 2026-03-06

**Authors:** Ningning Wang, Hongbin Yi, Jiale Yang, Aizezijiang Aierken, Haishaerjiang Wushouer, Sheng Han, Wenbing Yao

**Affiliations:** 1 Department of Pharmacy, Xinjiang Medical University, Urumqi, China; 2 International Research Centre for Medicinal Administration, Peking University, Beijing, China; 3 School of International Pharmaceutical Business, China Pharmaceutical University, Nanjing, China; 4 Department of Pharmacy, The First Affiliated Hospital of Xinjiang Medical University, Urumqi, China; 5 Xinjiang Key Laboratory of Clinical Drug Research, Urumqi, China; 6 Department of Pharmacy Administration and Clinical Pharmacy, School of Pharmaceutical Sciences, Peking University, Beijing, China

## Abstract

**Background:**

The high incidence and economic burden of coronary heart disease (CHD) represent a major public health challenge in Xinjiang, a region in northwest China. We aimed to assess the effects of a diagnosis-related group (DRG) policy on treatment costs and treatment efficiency in Bortala Mongol Autonomous Prefecture, Kashgar, and Kizilsu Kirghiz Autonomous Prefecture of Xinjiang, China.

**Methods:**

We retrieved data on CHD inpatients from three prefectures in the Xinjiang medical insurance database. Then, we used interrupted time series analyses to evaluate the effect of the DRG policy on these inpatients. The analysis spanned the period from 1 January 2022 to 31 December 2024, with the policy intervention point for each prefecture defined by its respective DRG implementation date. We divided patients into pre-DRG and post-DRG groups based on the timing of the DRG policy’s implementation. The outcome variables for treatment costs were total cost, drug cost, medical supplies cost, and out-of-pocket (OOP) payments; the outcome variable for treatment efficiency was length of stay (LOS).

**Results:**

Compared to the pre-DRG period, the total cost, drug cost, OOP, and LOS for CHD inpatients in three prefectures all decreased, with a growth rate (GR)<0. In contrast, the medical supplies cost in the Bortala Mongol Autonomous Prefecture and the Kizilsu Kirghiz Autonomous Prefecture increased (GR>0). Following the implementation of the DRG in Xinjiang, CHD inpatients in the Bortala Mongol Autonomous Prefecture saw a significant downward trend in drug cost (*β*_3_ = −26.898; 95% confidence interval (CI) = −45.303, −8.493; *P* = 0.004), and their LOS decreased significantly in both level (*P* = 0.001) and trend (*P* = 0.034). The total cost for CHD inpatients in Kashgar showed a significant downward trend, and drug cost decreased significantly in both level (*P* = 0.003) and trend (*P* < 0.001). However, we observed no statistically significant differences in the remaining indicators of the two regions, or for all changes for CHD inpatients in the Kizilsu Kirghiz Autonomous Prefecture (all *P* > 0.05).

**Conclusions:**

The DRG policy in Xinjiang has successfully controlled certain treatment costs and improved treatment efficiency for inpatients with CHD in the Bortala Mongol Autonomous Prefecture and in Kashgar. However, the implementation of DRG in the Kizilsu Kirghiz Autonomous Prefecture has not yet shown significant results and thus warrants monitoring and research.

Rapid increases in health care costs and unequal distribution of medical resources have become pervasive challenges for health systems globally in recent years [1]. Payment models based on diagnosis-related groups (DRGs) have emerged as possible solutions and have been adopted across numerous developed nations and regions [2,3]. By classifying inpatient episodes into standardised groups according to diagnosis, surgical interventions, patient demographics (*e.g.* age, gender), or comorbidities and complications, the DRG systems create case groups that are relatively homogeneous in terms of both resource consumption and clinical complexity [4]. In contrast to the conventional fee-for-service (FFS) approach, DRGs incentivise health care providers to contain costs without compromising quality. This is achieved through predetermined, group-specific payment rates that discourage overtreatment and superfluous services, thus fostering a more rational allocation of health resources [5]. International evidence from the USA, Germany, Australia, and many European countries demonstrates the successful integration of DRG-based payment into hospital financing systems [6-9]. This strategy has proven effective in improving health care efficiency and quality, while achieving substantial cost control [10].

China’s total national health expenditure has seen sustained growth, increasing from CNY 1.75 trillion in 2009 to 7.22 trillion in 2020, representing an average annual growth rate of 13.8% [11]. Currently, the country’s goal in reforming its health care security system is to enhance the quality of care, while curbing rising medical expenditures. The strategy to achieve this is to implement a multi-component payment system; China initiated an accelerated rollout of China’s healthcare security DRG pilot programmes in 2019, aiming to develop and implement a domestically tailored DRG classification system and corresponding payment standards [12].

While the impacts of DRG are well-documented in low- and middle-income countries (LMICs), China’s DRG reform is characterised by unique implementation, design, and governance features. Unlike fragmented systems elsewhere, the country operates a unified, state-led system serving over 1.4 billion people, dominated by public hospitals and driven by the strong administrative capacity of the National Healthcare Security Administration [13,14]. Rather than adopting international models like the All-Patient DRG, China developed the China National DRG, an algorithm optimised for local practices and regional disparities [15,16]. This algorithm highlights the feasibility of scaling payment systems in large populations and leveraging state capacity to accelerate reform, while balancing efficiency with universal health coverage goals [17].

A substantial body of evidence exists regarding the effectiveness of DRG policies in countries worldwide [9]. However, given the variations in demographic structures, socioeconomic development, and policy implementation across context, the performance of China’s DRG policy warrants further assessment. To date, numerous provinces across China have actively piloted and implemented the DRG payment policy, achieving initial successes in several key areas, such as establishing a standardised disease classification framework, developing grouping software, calculating payment tariffs, and enhancing the performance evaluation system [18–20]. However, empirical research on this topic remains scarce, largely because DRG policy was rolled out nationally in 2019. Specifically, there is a research gap on the DRG policy’s effect in less economically developed provinces, including Xinjiang, Tibet, and Inner Mongolia. The specificity of Xinjiang’s local disease spectrum and the substantial economic burden of high-incidence diseases, for example, shows a demand for long-term health care payment reform [21–23]. Following a successful pilot in Urumqi, Xinjiang expanded its DRG reform to 11 additional prefectures in 2022–2023, notably Kashgar, Bortala Mongol Autonomous Prefecture, and Kizilsu Kirghiz Autonomous Prefecture [24].

Xinjiang is currently facing a significant public challenge due to a high prevalence of coronary heart disease (CHD) and the ensuing economic burden. By utilising local medical insurance data, we wanted to assess the effects of the DRG policy on treatment costs and treatment efficiency of CHD inpatients in Bortala Mongol Autonomous Prefecture, Kashgar, and Kizilsu Kirghiz Autonomous Prefecture of Xinjiang, in order to offer evidence-based insights that would guide DRG policy-making and practice not only in the region, but also in underdeveloped areas across Western China.

## METHODS

In this quasi-experimental design study, we employed an interrupted time series (ITS) model to evaluate the changes in patient medical costs and treatment efficiency following the implementation of the DRG policy in the Bortala Mongol Autonomous Prefecture, Kashgar, and Kizilsu Kirghiz Autonomous Prefecture of Xinjiang. The observation period for the model was from 1 January 2022 to 31 December 2024, while the policy intervention time was determined based on the actual implementation dates of the DRG policy in each prefecture-level city of Xinjiang. Specifically, the start time for DRG policy was 1 May 2023 in Kashgar; 1 June 2023 in Bortala Mongol Autonomous Prefecture; and 1 July 2023 in Kizilsu Kirghiz Autonomous Prefecture [25–27]. We divided patients into a pre-DRG group and a post-DRG group based on the timing of DRG policy implementation in their respective regions. We report our findings per the STROBE guidelines [28].

### Data source and sample selected

We retrieved data from the medical insurance database of the Xinjiang, which contains health care data (patient demographics, front pages of medical records, detailed cost breakdowns) from all public hospitals in the region. To ensure sample homogeneity, we included hospitals in the Bortala Mongol Autonomous Prefecture, Kashgar, and Kizilsu Kirghiz Autonomous Prefecture only if they implemented the DRG policy in 2023, and those who did so in 2024 or at a later data.

We included patients hospitalised with a primary diagnosis of CHD (ICD-10 codes I20–I25) between 1 January 2022 and 31 December 2024 and patients who received corresponding treatment for CHD in hospitals within the Xinjiang Uygur Autonomous Region. We excluded patients missing key cost information and those with a length of stay (LOS) shorter than one day.

### Outcome variables

We selected four outcome variables from the database to evaluate the impact of DRG policy on medical costs and patients’ financial burden: total cost per inpatient, drug cost per inpatient, medical supplies cost per inpatient, and out-of-pocket (OOP) cost per inpatient. Among these, total cost per inpatient and OOP cost were considered the primary outcome variables. Given that the study period encompassed only one year before and after policy implementation, with some observations falling within the same year, we did not adjust cost data for inflation using the Consumer Price Index (CPI). Furthermore, we evaluated treatment efficiency by LOS, an additional outcome variable.

### Statistical analysis

We first conducted a descriptive analysis of the outcome variables for the three regions. Subsequently, we used one-way ANOVA to test whether there were significant differences in the means among the three regions.

Following this, we used an ITS model to evaluate the impact of the DRG payment policy on medical costs and treatment efficiency for inpatients with CHD. The model’s structure was as follows:

*Y_t_ = β*_0_ + *β*_1_*T_t_* + *β*_2_*X_t_* + *β*_3_*X_t_T_t_* + *ε_t_*

Here, *Y_t_* represents the aggregated outcome variable measured at each equally spaced time point *t*; *T_t_* denotes the time since the start of the study; *X_t_* is a dummy variable for the intervention (0 for the pre-intervention period, 1 otherwise); *X_t_T_t_* represents the interaction term; and *ε_t_* is the error term. In a single-group ITS model, indicates the intercept or initial level of the outcome variable; *β*_1_ represents the slope (or trend) of the outcome variable before the policy intervention; *β*_2_ indicates the immediate level change after the policy intervention; and *β*_3_ represents the difference in the slope (or trend) of the outcome before and after the intervention. Therefore, a statistically significant *β*_2_ (based on its *P*-value) indicates an immediate policy effect, while a statistically significant *β*_3_ indicates a policy effect that changes over time [29-31].

The total cost, drug cost medical supplies cost, OOP, and LOS for per inpatient served as the outcome variables (*Y_t_*) in the ITS models. We employed the Newey-West method for parameter estimation and standard error calculation based on a generalised linear model to address potential autocorrelation and heteroscedasticity [30]. We set the significance level at an α of 0.05, and reported the 95% confidence intervals (95% CIs) for the model estimates.

We processed all raw data using Microsoft Excel, version 2021 (Microsoft Corp, Washington State, USA). We performed the initial descriptive analyses using SPSS, version 27 (IBM, Armonk, New York, USA), and constructed all models using Stata, version 17 (StataCorp LLC, College Station, Texas, USA).

## RESULTS

Our analysis included 120 600 inpatients with CHD from medical insurance databases. Kashgar accounted for 34 541 inpatients in the pre-DRG period (1 January 2022 to 30 April 2023) and 59 690 inpatients in the post-DRG period (1 May 2023 to 31 December 2024). The Bortala Mongol Autonomous Prefecture contributed 4637 inpatients in the pre-DRG period (1 January 2022 to 31 May 2023) and 6187 inpatients in the post-DRG period (1 June 2023 to 31 December 2024). The Kizilsu Kirghiz Autonomous Prefecture had 7216 inpatients in the pre-DRG period (1 January 2022 to 30 June 2023) and 8329 inpatients in the post-DRG period (1 July 2023 to 31 December 2024).

### Descriptive analysis

Prior to DRG implementation, inpatients with CHD in the Bortala Mongol Autonomous Prefecture had the highest OOP cost (CNY 2372.036), inpatients with CHD in Kashgar had the highest drug cost (CNY 1650.861) and the longest LOS (7.469 days), and inpatients with CHD in the Kizilsu Kirghiz Autonomous Prefecture had the highest total cost (CNY 9735.641) and medical supplies cost (CNY 2392.38). We generally observed a downward trend in total costs, drug costs, OOP costs, and LOS among the inpatients with CHD in all three prefectures after DRG implementation. Kashgar saw the most significant reduction in total cost, with a growth rate (GR) of −20.238%. However, medical supplies costs showed varying trends across prefectures: they increased in the Bortala Mongol Autonomous Prefecture (GR = 34.783%) and the Kizilsu Kirghiz Autonomous Prefecture (GR = 7.852%), but decreased in Kashgar (GR = −9.684%). Drug costs saw the largest reduction in all three prefectures, with GRs of −32.755% in the Bortala Mongol Autonomous Prefecture, −34.969% in Kashgar, and −24.359% in the Kizilsu Kirghiz Autonomous Prefecture (**Table 1**).

**Table 1:** Changes in treatment cost and efficiency in three prefectures of Xinjiang before and after the implementation of DRG

Outcome variables by prefecture	Pre-DRG	*P*-value	Post-DRG	*P*-value	GR
Total costs in CNY		<0.001		<0.001	
*Bortala Mongol Autonomous Prefecture*	7620.461		7468.678		−1.992%
*Kashgar*	8572.735		6837.768		−20.238%
*Kizilsu Kirghiz Autonomous Prefecture*	9735.641		8974.003		−7.823%
Drug costs in CNY		<0.001		<0.001	
*Bortala Mongol Autonomous Prefecture*	1033.121		694.719		−32.755%
*Kashgar*	1650.861		1073.568		−34.969%
*Kizilsu Kirghiz Autonomous Prefecture*	1616.383		1222.65		−24.359%
Medical supplies costs in CNY		<0.001		<0.001	
*Bortala Mongol Autonomous Prefecture*	1648.681		2222.147		34.783%
*Kashgar*	1188.689		1073.582		−9.684%
*Kizilsu Kirghiz Autonomous Prefecture*	2392.38		2580.224		7.852%
OOP in CNY		<0.001		<0.001	
*Bortala Mongol Autonomous Prefecture*	2372.036		2023.841		−14.679%
*Kashgar*	1642.327		1275.721		−22.322%
*Kizilsu Kirghiz Autonomous Prefecture*	2036.122		2025.458		−0.524%
LOS in days		<0.001		<0.001	
*Bortala Mongol Autonomous Prefecture*	6.776		5.833		−13.917%
*Kashgar*	7.469		6.836		−8.475%
*Kizilsu Kirghiz Autonomous Prefecture*	7.247		6.877		−5.106%

DRG – diagnosis-related group, GR – growth rate, LOS – length of stay, OOP – out-of-pocket

### Changes in treatment costs and efficiency in the Bortala Mongol Autonomous Prefecture

**Figure 1:**
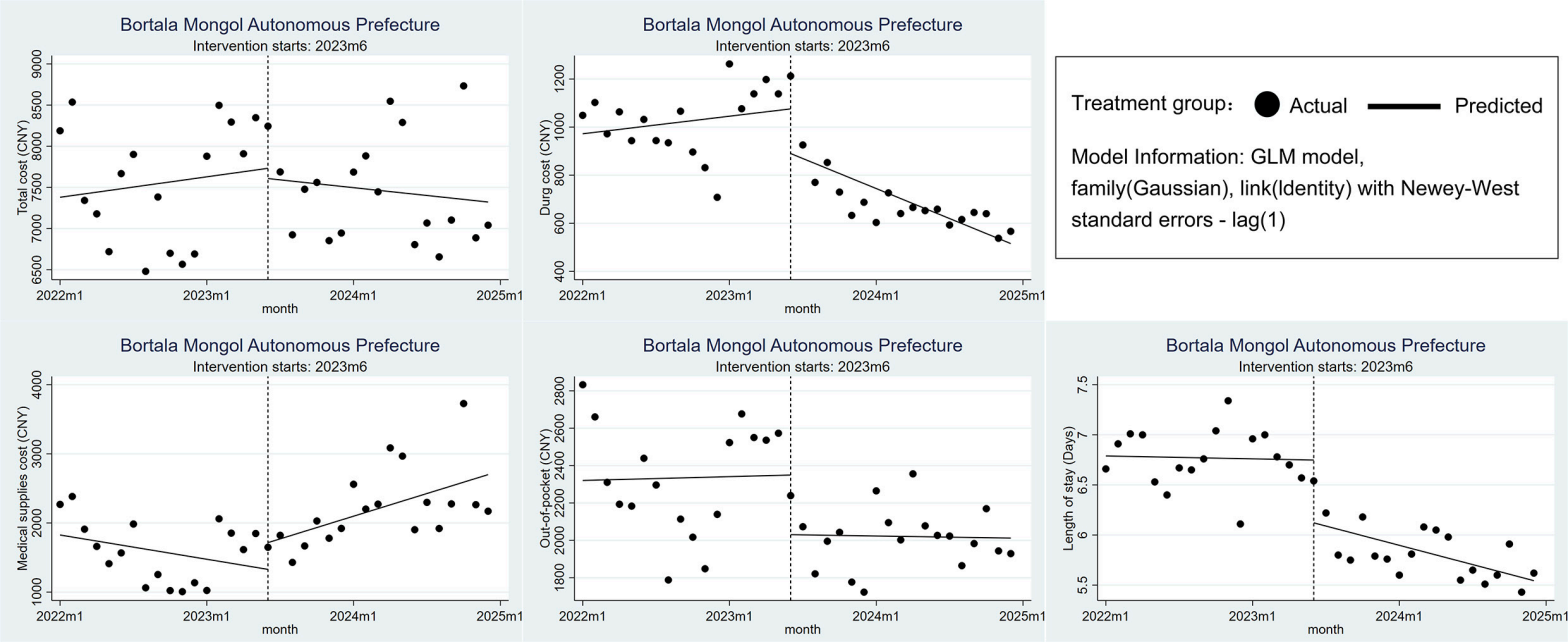
Changes in treatment costs and efficiency in the Bortala Mongol Autonomous Prefecture.

After the implementation of DRG, both the level and trend of total costs and OOP costs for CHD inpatients in the Bortala Mongol Autonomous Prefecture showed a decrease, but these changes lacked statistical significance (*P* > 0.05). Although the decrease in the level of drug cost for CHD inpatients was not statistically significant, we observed a significant downward trend in drug cost after the DRG implementation (*β*_3_ = −26.898; 95% CI = −45.303, −8.493; *P* = 0.004). The LOS for CHD inpatients was also significantly reduced by 0.627 days (*β*_2_ = −0.627; 95% CI = −0.99, −0.263; *P* = 0.001), with a significant downward trend after DRG implementation (*β*_3_ = −0.03; 95% CI = −0.057, −0.002; *P* = 0.034). In contrast, medical supplies costs increased post-DRG implementation, with a statistically significant trend (*β*_3_ = 83.9; 95% CI = 19.421, 148.38; *P* = 0.011) (**Figure 1**, **Table 2**; Table S1 in the **Online Supplementary Document**).

**Table 2:** Statistical results of ITS for treatment costs and efficiency for inpatients with CHD before and after the implementation of DRG

	Coefficient estimates (95% CI)			
**Outcome variables by prefecture**	**Level change (*β*_2_)**	***P*-value**	**Trend change (*β*_3_)**	***P*-value**
Bortala Mongol Autonomous Prefecture				
*Total costs in CNY*	−124.496 (−1069.777, 820.785)	0.796	−36.545 (−137.544, 64.454)	0.478
*Drug costs in CNY*	−185.703 (−398.988, 27.582)	0.088	−26.898 (−45.303, −8.493)	0.004
*Medical supplies costs in CNY*	386.482 (−250.294, 1023.258)	0.234	83.9 (19.421, 148.38)	0.011
*OOP in CNY*	−319.139 (−706.246, 67.969)	0.106	−2.717 (−42.377, 36.944)	0.893
*LOS in days*	−0.627 (−0.99, −0.263)	0.001	−0.03 (−0.057, −0.002)	0.034
Kashgar				
*Total costs in CNY*	−503.886 (−1310.076, 302.303)	0.221	−110.269 (−182.048, −38.489)	0.003
*Drug costs in CNY*	−309.96 (−517.592, −102.328)	0.003	−46.317 (−63.662, −28.972)	<0.001
*Medical supplies costs in CNY*	50.038 (−230.489, 330.565)	0.727	−0.107 (−25.297, 25.083)	0.993
*OOP in CNY*	28.743 (−214.596, 272.083)	0.817	−4.9 (−25.485, 15.685)	0.641
*LOS in days*	−0.348 (−0.769, 0.073)	0.105	−0.02 (−0.05, 0.01)	0.192
Kizilsu Kirghiz Autonomous Prefecture				
*Total costs in CNY*	−1003.413 (−2595.141, 588.315)	0.217	20.341 (−102.839, 143.521)	0.746
*Drug costs in CNY*	−255.483 (−555.192, 44.226)	0.095	−13.445 (−38.458, 11.568)	0.292
*Medical supplies costs in CNY*	−326.961 (−1231.729, 577.807)	0.479	−20.003 (−84.35, 44.343)	0.542
*OOP in CNY*	156.456 (−111.489, 424.4)	0.252	2.968 (−22.512, 28.448)	0.819
*LOS in days*	−0.279 (−0.741, 0.183)	0.236	−0.014 (−0.056, 0.028)	0.521

CI – confidence interval, LOS – length of stay, OOP – out-of-pocket

### Changes in treatment costs and efficiency in Kashgar

**Figure 2:**
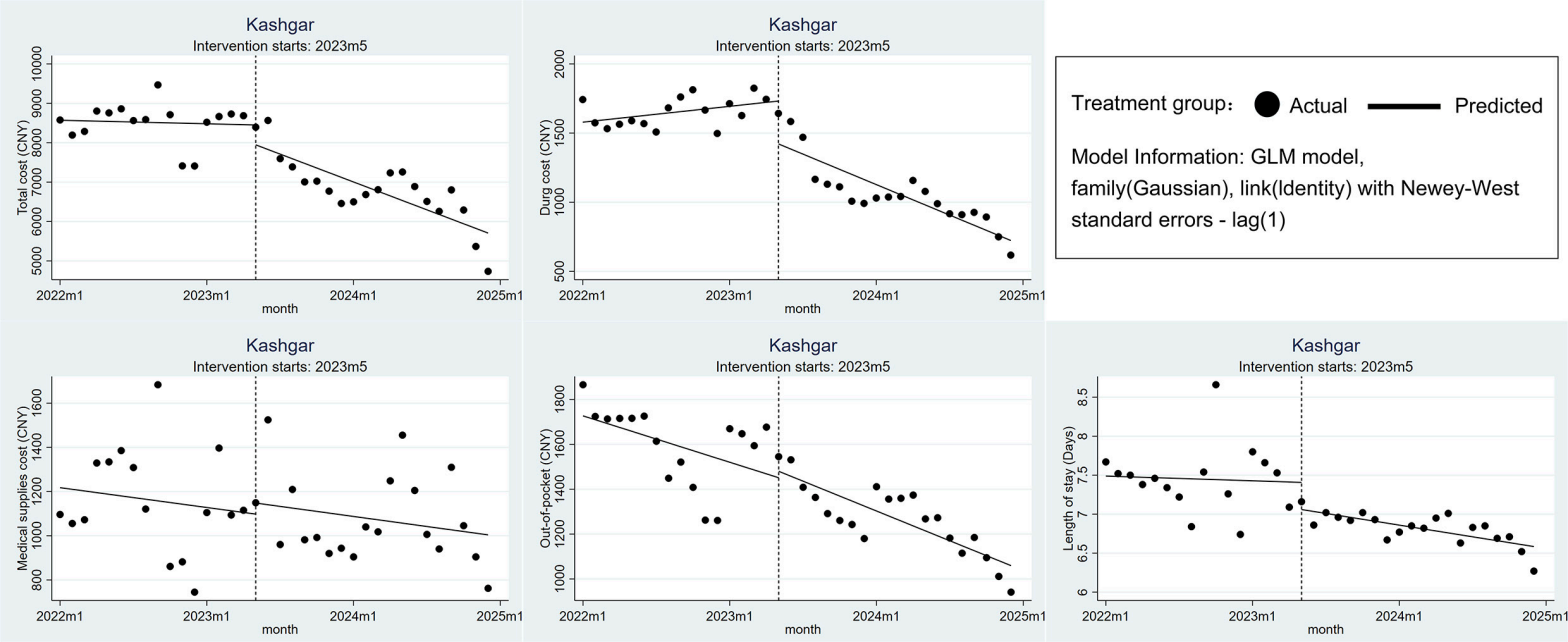
Changes in treatment costs and efficiency in Kashgar.

Following the implementation of the DRG policy, the total cost for CHD inpatients in Kashgar decreased, with a significant downward trend (*β*_3_ = −110.269; 95% CI = −182.048, −38.489; *P* = 0.003). The drug cost for these CHD inpatients saw an immediate level decrease of 309.96 CNY (*β*_2_ = −309.96; 95% CI = −517.592, −102.328; *P* = 0.003) and a significant downward trend (*β*_3_ = −46.317; 95% CI = −63.662, −28.972; *P* < 0.001). In the month of the DRG policy implementation, the medical supplies cost and OOP cost for Kashgar’s hospitalised CHD patients fluctuated slightly, while the LOS showed a minor reduction. After its implementation, medical supplies cost, OOP, and LOS all exhibited a downward trend, but these changes were not statistically significant (*P* > 0.05) (**Figure 2**, **Table 2**; Table S2 in the **Online Supplementary Document**).

### Changes in treatment costs and efficiency in the Kizilsu Kirghiz Autonomous Prefecture

**Figure 3:**
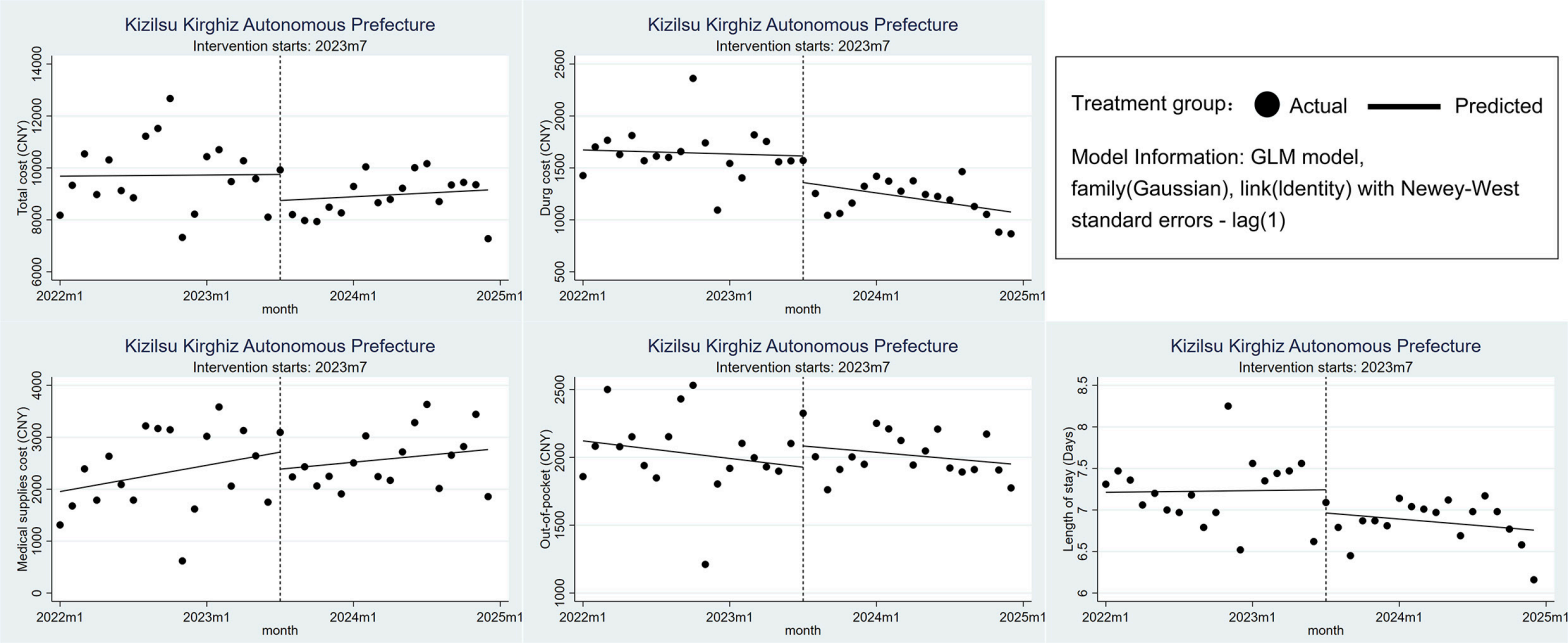
Changes in treatment costs and efficiency in the Kizilsu Kirghiz Autonomous Prefecture.

The initial month of the DRG policy saw reductions in total costs, drug costs, medical supplies costs, and LOS for CHD inpatients, excluding OOP. However, these reductions lacked statistical significance (*P* > 0.05). Following the implementation of the DRG, we noted an increase in the long-term trend for total costs, although its level remained lower than the pre-DRG implementation period. In contrast, the long-term trends for drug costs, medical supplies costs, and LOS were all downward. Compared to its pre-DRG implementation trend, the downward trend of OOP decelerated. Similar to the level changes, these trend changes were also not statistically significant (*P* > 0.05) (**Figure 3**, **Table 2**; Table S3 in the **Online Supplementary Document**).

## DISCUSSION

Our results indicate that the DRG policy in Xinjiang has only partially controlled certain treatment costs (reduction of total costs, drug costs and OOP costs) and improved treatment efficiency (reduction of LOS) for inpatients with CHD in the Bortala Mongol Autonomous Prefecture and Kashgar. They thus offer important data for advancing health care reform and refining policy design.

One of our key findings is that the effects of the DRG policy show significant regional heterogeneity. Kashgar, which has the most extensive health care infrastructure and the highest pre-reform cost levels among the sampled regions, experienced the most pronounced improvements under the reform: total costs showed a significant downward trend and drug costs decreased significantly in both level and trend, effectively enhancing the affordability of inpatients with CHD in Kashgar. This is highly consistent with numerous domestic and international studies indicating that DRG payment can effectively control the rapid growth of medical costs [9,32–35]. However, the impact on CHD inpatients in the Bortala Mongol Autonomous Prefecture and the Kizilsu Kirghiz Autonomous Prefecture differed somewhat from that in Kashgar. While total costs for CHD inpatients in Boratala did not decrease significantly, LOS was substantially reduced. These results are also consistent with those of previous studies [36], suggesting that DRG policy in Bortala was more effective in optimising clinical pathways and accelerating bed turnover, thereby significantly improving the efficiency of medical resource utilisation for CHD inpatients. Although four out of five outcome variables related to treatment costs and efficiency showed improvement in Kizilsu, none of these changes were statistically significant, implying that the policy has no impact on these variables. These variations in outcomes clearly indicate that DRG is not a standardised, one-size-fits-all solution [37]. Its policy effectiveness appears to be closely associated with factors such as regional economic development, hospital management capacity, and regulatory environments. This finding suggests that health policymakers should prioritise setting region-specific goals based on local core challenges when designing and implementing DRG policies, and adopt more refined, differentiated implementation strategies.

Our results are indicative of the cost structure shifting in hospitals in the Bortala Mongol Autonomous Prefecture and the Kizilsu Kirghiz Autonomous Prefecture following the implementation of DRG. For instance, the key reason why the total costs for CHD inpatients in these two regions did not decrease significantly lies in the substantial increase in medical supplies costs after DRG was introduced. This uncovers a potential risk: when China’s DRG policy imposes rigid constraints on drug costs and LOS, hospitals may adjust their cost structures by shifting profit centres from pharmaceuticals to medical supplies in order to maintain revenue levels. Such cost-shifting behaviour partially offsets the cost-containment effects of DRG, rendering the reduction in total cost statistically insignificant. This issue warrants close attention and resolution in future policy optimisation, with our findings offering compelling explanation for the inconsistent cost-control outcomes observed in previous DRG-related studies and providing a valuable reference for policymakers [38]. Future DRG reforms should evolve beyond a narrow focus on ‘cost control’ to embrace comprehensive ‘cost management’. This requires establishing an integrated oversight system that encompasses all health care service items, including pharmaceuticals, medical supplies, and examinations, thereby preventing hospitals from circumventing policy constraints through internal cost restructuring.

While the widespread reduction about LOS in the Bortala Mongol Autonomous Prefecture could be seen as a positive signal of improved treatment efficiency, we must be aware of the potential risks to medical quality that may underlie this gain. The inherent incentive mechanism of the DRG payment system, characterised by ‘retaining surpluses and bearing deficits’, may induce hospitals to engage in moral hazard. For instance, hospitals might reduce costs by cutting necessary examinations, prematurely discharging CHD inpatients, or selectively admitting less severe CHD cases. While such behaviour can boost treatment efficiency for CHD inpatients in the short term, it may lead to adverse consequences, including increased readmission rates and suboptimal treatment outcomes [39]. Previous studies have indicated that if physicians face greater financial pressure in the future, the quality of care could be severely compromised [39,40]. Therefore, whether the observed reduction in LOS in this study stems from genuine optimisation of clinical processes or from a potential degradation in the quality of medical services remains to be evaluated. Such assessments require longer-term data incorporating patient outcome indicators, such as readmission rates, complication rates, mortality, and patient satisfaction. This underscores the urgency of deeply integrating a medical quality evaluation system with the DRG payment system, ensuring that the DRG reform consistently advances toward its core goal: providing patients with the best possible health outcomes at a reasonable cost.

Regarding the results for the Kizilsu Kirghiz Autonomous Prefecture, which non-significant changes, it would be premature to conclude that the DRG policy has been ineffective for inpatients with CHD. Our descriptive analysis indicates that four out of five outcome variables in the prefecture showed positive changes, suggesting that the DRG policy has already exerted a preliminary, positive guiding effect. The non-significance of these changes may be attributed to the region’s relatively weak health care infrastructure, the short duration of policy implementation, or the limited capacity of hospitals to adapt to the new payment system. Future research could explore why the DRG seems to be effective in some regions and less significant in others, and should guide corresponding solutions or policies based on local conditions.

### Limitations

This study has several limitations. First, due to a lack of data, we did not perform baseline adjustment based on demographic variables and exclusively selected inpatients with CHD from the Bortala Mongol Autonomous Prefecture, Kashgar, and the Kizilsu Kirghiz Autonomous Prefecture. Consequently, the generalisability of our findings to other prefectures in Xinjiang requires further validation. Second, while we focused on outcome variables such as treatment costs and efficiency, we did not incorporate key clinical endpoints, including readmission rates, complication rates, and mortality. Therefore, the long-term impact of the DRG policy on treatment outcomes for CHD patients should be investigated further by integrating such patient outcome data. Finally, due to the extremely small number of CHD cases in hospitals within these three prefectures that had not implemented the DRG policy, establishing a valid control group was infeasible. This precludes the full exclusion of potential confounding factors. Future studies should employ more rigorous causal inference methods, such as matching patients based on baseline characteristics, to derive more reliable conclusions.

## CONCLUSIONS

The DRG policy in Xinjiang has successfully controlled certain treatment costs and improved treatment efficiency for inpatients with CHD in the Bortala Mongol Autonomous Prefecture and Kashgar. However, the implementation of DRG policy has shown regional heterogeneity and has led to cost structure shifting. To address this, health policymakers should transition from a narrow focus on cost control to a more robust approach of cost management. It is essential to establish an integrated oversight system for all medical service items to prevent hospitals from circumventing policy constraints through internal cost restructuring.

## Additional material


Online Supplementary Document
