# Supplementary material for: The effect of diagnosis-related group policy on treatment cost and treatment efficiency of inpatients with coronary heart disease in Xinjiang: an interrupted time series analysis
Source: J Glob Health. 2026 Mar 6;16:04078. doi: 10.7189/jogh.16.04078 (PMC12964326; doi:10.7189/jogh.16.04078)
Supplement: Online Supplementary Document [file jogh-16-04078-s001.pdf]

Supplement to: Wang N, Yi H, Yang J, Aierken A, Wushouer H, Han S, Yao W. The effect of diagnosis-related group policy on treatment cost and treatment efficiency of inpatients with coronary heart disease in Xinjiang: an interrupted time series analysis. J Glob Health. 2026;16:04078.

Supplementary materials

Table S1 ITS statistical results Analysis of Treatment Costs and Efficiency Pre- and Post-DRG Policy Implementation in Bortala Mongol Autonomous Prefecture 2

Table S2 ITS statistical results Analysis of Treatment Costs and Efficiency Pre- and Post-DRG Policy Implementation in Kashgar 3

Table S3 ITS statistical results Analysis of Treatment Costs and Efficiency Pre- and Post-DRG Policy Implementation in Kizilsu Kirghiz Autonomous Prefecture 4

Table S1 ITS statistical results Analysis of Treatment Costs and Efficiency Pre- and Post-DRG Policy Implementation in Bortala Mongol Autonomous Prefecture

Outcome variables	Coefficients	Estimates	Std. Error	z value	P value	95% CI
Total cost (CNY)	β_0	7380.385	410.744	17.97	0	6575.342 to 8185.428
	β_1	20.708	43.473	0.48	0.634	-64.497 to 105.913
	β_2	-124.496	482.295	-0.26	0.796	-1069.777 to 820.785
	β_3	-36.545	51.531	-0.71	0.478	-137.544 to 64.454
Durg cost (CNY)	β_0	972.205	44.36	21.92	0	885.26 to 1059.149
	β_1	6.096	6.252	0.98	0.33	-6.158 to 18.35
	β_2	-185.703	108.821	-1.71	0.088	-398.988 to 27.582
	β_3	-26.898	9.391	-2.86	0.004	-45.303 to -8.493
Medical supplies cost (CNY)	β_0	1826.651	234.251	7.8	0	1367.528 to 2285.775
	β_1	-29.245	26.915	-1.09	0.277	-81.997 to 23.508
	β_2	386.482	324.892	1.19	0.234	-250.294 to 1023.258
	β_3	83.9	32.898	2.55	0.011	19.421 to 148.38
Out-of-pocket (CNY)	β_0	2320.505	183.433	12.65	0	1960.982 to 2680.028
	β_1	1.712	18.818	0.09	0.928	-35.171 to 38.595
	β_2	-319.139	197.507	-1.62	0.106	-706.246 to 67.969
	β_3	-2.717	20.235	-0.13	0.893	-42.377 to 36.944
Length of stay (Days)	β_0	6.789	0.111	61.25	0	6.572 to 7.007
	β_1	-0.002	0.011	-0.23	0.821	-0.023 to 0.019
	β_2	-0.627	0.186	-3.38	0.001	-0.99 to -0.263
	β_3	-0.03	0.014	-2.12	0.034	-0.057 to -0.002

Table S2 ITS statistical results Analysis of Treatment Costs and Efficiency Pre- and Post-DRG Policy Implementation in Kashgar

Outcome variables	Coefficients	Estimates	Std. Error	z value	P value	95% CI
Total cost (CNY)	β_0	8571.532	155.875	54.99	0	8266.022 to 8877.043
	β_1	-7.57	23.452	-0.32	0.747	-53.535 to 38.394
	β_2	-503.886	411.329	-1.23	0.221	-1310.076 to 302.303
	β_3	-110.269	36.623	-3.01	0.003	-182.048 to -38.489
Durg cost (CNY)	β_0	1578.741	48.689	32.43	0	1483.313 to 1674.168
	β_1	9.557	5.184	1.84	0.065	-0.604 to 19.718
	β_2	-309.96	105.936	-2.93	0.003	-517.592 to -102.328
	β_3	-46.317	8.85	-5.23	0	-63.662 to -28.972
Medical supplies cost (CNY)	β_0	1217.619	89.351	13.63	0	1042.495 to 1392.743
	β_1	-7.476	10.302	-0.73	0.468	-27.667 to 12.716
	β_2	50.038	143.129	0.35	0.727	-230.489 to 330.565
	β_3	-0.107	12.852	-0.01	0.993	-25.297 to 25.083
Out-of-pocket (CNY)	β_0	1726.99	54.856	31.48	0	1619.474 to 1834.505
	β_1	-17.236	9.576	-1.8	0.072	-36.005 to 1.532
	β_2	28.743	124.155	0.23	0.817	-214.596 to 272.083
	β_3	-4.9	10.503	-0.47	0.641	-25.485 to 15.685
Length of stay (Days)	β_0	7.489	0.102	73.64	0	7.289 to 7.688
	β_1	-0.005	0.014	-0.36	0.722	-0.033 to 0.023
	β_2	-0.348	0.215	-1.62	0.105	-0.769 to 0.073
	β_3	-0.02	0.015	-1.3	0.192	-0.05 to 0.01

Table S3 ITS statistical results Analysis of Treatment Costs and Efficiency Pre- and Post-DRG Policy Implementation in Kizilsu Kirghiz Autonomous Prefecture

Outcome variables	Coefficients	Estimates	Std. Error	z value	P value	95% CI
Total cost (CNY)	β_0	9681.49	506.353	19.12	0	8689.057 to 10673.92
	β_1	3.677	48.727	0.08	0.94	-91.827 to 99.18
	β_2	-1003.413	812.121	-1.24	0.217	-2595.141 to 588.315
	β_3	20.341	62.848	0.32	0.746	-102.839 to 143.521
Durg cost (CNY)	β_0	1672.995	70.212	23.83	0	1535.382 to 1810.608
	β_1	-3.241	7.027	-0.46	0.645	-17.013 to 10.531
	β_2	-255.483	152.916	-1.67	0.095	-555.192 to 44.226
	β_3	-13.445	12.762	-1.05	0.292	-38.458 to 11.568
Medical supplies cost (CNY)	β_0	1954.485	249.165	7.84	0	1466.13 to 2442.84
	β_1	42.165	27.465	1.54	0.125	-11.665 to 95.995
	β_2	-326.961	461.625	-0.71	0.479	-1231.729 to 577.807
	β_3	-20.003	32.83	-0.61	0.542	-84.35 to 44.343
Out-of-pocket (CNY)	β_0	2121.307	108.378	19.57	0	1908.889 to 2333.725
	β_1	-10.777	8.851	-1.22	0.223	-28.126 to 6.572
	β_2	156.456	136.709	1.14	0.252	-111.489 to 424.4
	β_3	2.968	13	0.23	0.819	-22.512 to 28.448
Length of stay (Days)	β_0	7.212	0.119	60.52	0	6.979 to 7.446
	β_1	0.002	0.014	0.12	0.907	-0.027 to 0.03
	β_2	-0.279	0.236	-1.18	0.236	-0.741 to 0.183
	β_3	-0.014	0.022	-0.64	0.521	-0.056 to 0.028
